# Application of a deep learning algorithm for detection and visualization of hip fractures on plain pelvic radiographs

**DOI:** 10.1007/s00330-019-06167-y

**Published:** 2019-04-01

**Authors:** Chi-Tung Cheng, Tsung-Ying Ho, Tao-Yi Lee, Chih-Chen Chang, Ching-Cheng Chou, Chih-Chi Chen, I-Fang Chung, Chien-Hung Liao

**Affiliations:** 1 Department of Trauma and Emergency Surgery, Chang Gung Memorial Hospital, Linkou, Chang Gung University, Taoyuan, Taiwan; 2 Institute of Biomedical Informatics, National Yang-Ming University, Taipei, Taiwan; 3 Departments of Nuclear Medicine and Molecular Imaging Center, Chang Gung Memorial Hospital, Linkou, Chang Gung University, Taoyuan, Taiwan; 4 Donald Bren School of Information and Computer Sciences, University of California Irvine, Irvine, CA USA; 5 Department of Medical Imaging and Intervention, Chang Gung Memorial Hospital, Linkou, Chang Gung University, Taoyuan, Taiwan; 6 Departments of Rehabilitation and physical medicine, Chang Gung Memorial Hospital, Linkou, Chang Gung University, Taoyuan, Taiwan; 7 Center for Systems and Synthetic Biology, National Yang-Ming University, Taipei, Taiwan; 8 Preventive Medicine Research Center, National Yang-Ming University, Taipei, Taiwan; 9 Center for Artificial Intelligence in Medicine, Chang Gung Memorial Hospital, Linkou, Taoyuan, Taiwan

**Keywords:** Hip fractures, Neural network (computer), Machine learning, Algorithms

## Abstract

**Objective:**

To identify the feasibility of using a deep convolutional neural network (DCNN) for the detection and localization of hip fractures on plain frontal pelvic radiographs (PXRs).

**Summary of background data:**

Hip fracture is a leading worldwide health problem for the elderly. A missed diagnosis of hip fracture on radiography leads to a dismal prognosis. The application of a DCNN to PXRs can potentially improve the accuracy and efficiency of hip fracture diagnosis.

**Methods:**

A DCNN was pretrained using 25,505 limb radiographs between January 2012 and December 2017. It was retrained using 3605 PXRs between August 2008 and December 2016. The accuracy, sensitivity, false-negative rate, and area under the receiver operating characteristic curve (AUC) were evaluated on 100 independent PXRs acquired during 2017. The authors also used the visualization algorithm gradient-weighted class activation mapping (Grad-CAM) to confirm the validity of the model.

**Results:**

The algorithm achieved an accuracy of 91%, a sensitivity of 98%, a false-negative rate of 2%, and an AUC of 0.98 for identifying hip fractures. The visualization algorithm showed an accuracy of 95.9% for lesion identification.

**Conclusions:**

A DCNN not only detected hip fractures on PXRs with a low false-negative rate but also had high accuracy for localizing fracture lesions. The DCNN might be an efficient and economical model to help clinicians make a diagnosis without interrupting the current clinical pathway.

**Key Points:**

• *Automated detection of hip fractures on frontal pelvic radiographs may facilitate emergent screening and evaluation efforts for primary physicians.*

*• Good visualization of the fracture site by Grad-CAM enables the rapid integration of this tool into the current medical system.*

*• The feasibility and efficiency of utilizing a deep neural network have been confirmed for the screening of hip fractures*.

## Introduction

Hip fractures and the resulting postsurgical outcomes are significant public health concerns worldwide [1–5]. Hip fracture is a predominant injury in elderly patients, and the incidence is approximately 250,000 per year in the USA and is expected double in 30 years [1, 3, 6, 7]. Although the incidence has declined recently [3, 8], the number of hip fractures will increase due to the prolonged human life span and growing elderly population. As many as 20–30% of people with a hip fracture will die in the subsequent year [5, 9–11], and many will experience significant functional loss [5, 12]. Early diagnosis and management might preserve not only the function of the joint but also the ambulation and quality of life of the patient [13]. Frontal pelvic radiographs (PXRs) are an essential and widely used tool for image evaluation for hip fractures. However, the sensitivity of PXRs for assessing hip fracture is not optimal. Some previous studies showed that initial misdiagnosis was as high as 7–14% [14, 15], and delayed diagnosis and treatment worsen the prognosis [16]. To avoid further health sequelae and medical costs associated with a delayed diagnosis, additional radiographs, nuclear medicine bone scans, computed tomography (CT) scans, and magnetic resonance imaging (MRI) scans have been recommended as routine diagnostics [14, 17]. However, it is not an effective, efficient, or economical method to use these diagnostic tools in routine examinations.

Digital medical imaging systems offer not only immediate and remote access [18] but also the possibility for computer-aided diagnostic procedures [19]. Computerized analysis based on deep learning has shown potential benefits as a diagnostic strategy and has recently become feasible [20]. The application and achievement of deep convolutional neural networks (DCNNs) in the medical field are expected to grow rapidly, and some studies have presented a great opportunity to apply deep learning in trauma [21–24]. DCNN has a proven ability to classify the bone structure of body parts and identify fractures in specific sites with expert-level accuracy [25–28]. Meanwhile, the efficiency and feasibility of using a DCNN for detecting hip fractures have not been evaluated completely. Although one recent article achieved high accuracy in identifying hip fractures using the region base method [29], the “black box” mechanism of deep learning is still the major hindrance for its clinical application. Many methods for the visual explanation of a DCNN have been developed, such as saliency mapping, class activation mapping (CAM), and gradient-weighted class activation mapping (Grad-CAM) [30]. Visualization mapping of images may validate the lesion detection ability of deep learning algorithms. Grad-CAM with the Keras-vis library generates a heatmap that visualizes the class-discriminative regions. In a medical image, Grad-CAM can help the physician to identify the pathologic region and validate the DCNN performance. Automated detection of hip fractures has potential benefits, such as increasing efficiency, decreasing misdiagnosis, reducing delayed management, and improving patient outcomes, especially in emergent situations if the algorithm achieved expert-level accuracy.

In this study, we developed an automated fracture diagnosis algorithm trained based on the DCNN to examine PXRs and investigated the performance compared with that of the physicians. We also investigated the validity of this algorithm by lesion visualization using Grad-CAM.

## Materials and methods

### Study population

We utilized the Chang Gung Trauma Registry Program (CGTRP) in Chang Gung Memorial Hospital (CGMH), Linkou, Taiwan. Demographic data, medical data, perioperative procedures, hospital procedures, medical imaging findings, follow-up data, and information regarding complications were recorded prospectively in a computerized database. We extracted the data and images of all trauma patients treated from August 2008 to December 2017 at CGMH, which is a level I trauma center. The Internal Review Board of CGMH approved the study.

### Training PXR dataset (2012–2016 PXR dataset)

Patients in the trauma registry treated from January 2012 to December 2016 were evaluated and selected if admitted and had frontal PXRs performed on the date of injury. The PXRs were stored automatically with a Python script for a picture archiving and communication system (PACS) viewer. The size of stored images varies from 2128 × 2248 pixels to 2688 × 2688 pixels, and the color is 8-bit grayscale. Each was given a serial number and de-identified in both the images and registry. After the images were stored, all images were evaluated. All labels in each image were carefully examined and removed.

### Limb radiograph dataset

The limb radiograph dataset, which included views of the ankles, feet, knees, wrists, and elbows, was collected and de-identified in a similar way as previously described. Each image was labeled as the corresponding body part during the automatic storage process. This dataset was used mainly to develop a pretrained medical imaging model for further transfer learning.

### Independent testing PXR dataset (2017 PXR dataset)

Another hundred patients were identified from the trauma registry during 2017 who had PXRs performed. There were 25 patients with femoral neck fractures, 25 with intertrochanteric fractures, and 50 without hip fractures. The PXRs performed on the date of injury were prepared as an independent testing dataset.

### Image labeling and preprocessing

After the 2008–2016 PXR datasets were established, the images were initially labeled as a hip fracture or no hip fracture according to the diagnosis in the trauma registry. The radiologist’s report, diagnosis, clinical course, and other related images, such as CT or other views of the hip joint, were reviewed if the label was questionable. Poor-quality images such as those with poor image contrast, positioning errors, and foreign body interference and those with other fractures, including femoral shaft fractures and pelvic fractures, were excluded. Each image was reviewed by a trauma surgeon for the preciseness of the label and quality of the images. The limb datasets were labeled automatically as previously described.

### Development of the algorithm

DCNNs are widely used in computer vision and medical image recognition [31]. A DCNN is a machine learning algorithm developed from an artificial neural network. The basic concept is to use pixel values from a digital image as inputs using techniques, such as convolution and pooling, on each layer and to adjust the weights in the neural network according to the difference between the output and true label. After a significant amount of imaging input is used as the training material, the weights in the neural network are adjusted to fit the problem. We used DenseNet-121 as the structure of our neural network [32]. The structure contains a dense block with a skip connection designed within the dense block. The input images were resized to 512 × 512 pixels with an 8-bit grayscale color to reduce the complexity and computation. Most studies use ImageNet as a pretraining material for “transfer learning” [26, 28, 33, 34]. Instead, we used the limb dataset as our pretraining material. The model was initially trained to identify the body part in view on each limb radiograph. We randomly chose 90% of the limb dataset for training and 10% for validation. The pretrained weights of the DCNN were preserved for PXR training. The PXR dataset was separated as 80% for training and 20% for validation. During the training process, image augmentation was applied with a zoom of 10%, horizontal flip, vertical flip, and rotation of 10°. The batch size was 8, and the Adam optimizer was used. The initial learning rate was 10^−3^ with a reduced learning rate on the plateau. The final model was trained with 60 epochs under the above hyperparameters.

### Evaluating the algorithm

The trained hip model was tested with the independent 2017 PXR dataset to evaluate its accuracy for identifying hip fractures. The probability generated by the model of hip fracture was evaluated with a receiver operating characteristic (ROC) curve and the area under the curve (AUC). A confusion matrix was also calculated using a cutoff level of probability 0.5 of hip fracture. For those PXR models that predict fractures, we also used a Grad-CAM to generate a heatmap that the model activated for the hip fracture to provide evidence that the model indeed recognized the fracture site. The heatmaps were also reviewed by a radiologist to compare with the fracture site on the original image to evaluate the ability of localization. We also recruited experts from the surgical, orthopedics, emergency, and radiology departments to evaluate the accuracy of each subspecialist. A web-based questionnaire for the 2017 PXR dataset was designed with a multiple-choice question to answer whether or not there was a hip fracture in the image.

### Statistical analysis and software

The software used to build the DCNN was based on an Ubuntu 14.04 operating system with TensorFlow 1.5.1 and Keras 2.1.4 open-source library with Python 3.6.5 (Python Software Foundation). The training process was run on an Intel® Core™ i7-7740X CPU 4.30 GHz with GeForce® GTX 1080 Ti GPU. All statistical analyses were performed using R 3.4.3 with extension packages “pROC,” “epiR,” “Publish,” and “ggplot2.” Continuous variables were evaluated with Student’s *t* test, and categorical variables were evaluated with the chi-square test. We compared the hip model and specialists using the sensitivity, specificity, false-negative rate, and false-positive rate, and the F1 scores and 95% confidence intervals (CIs) were calculated. ROC curves and AUCs were used to evaluate the performance of the model.

## Results

The demographic data of the patients in the 2008–2016 PXR dataset are shown in Table 1. We obtained 3605 PXRs to build the model and collected 25,505 different limb radiograph views from 12,583 patients, including radiographs of 6019 ankles, 3832 elbows, 4134 feet, and 3378 wrists.

**Table 1:** The demographic data of the frontal pelvic radiograph dataset

	With hip fracture	Without hip fracture	*p* value
Number of patients	1975	1630	
Age, mean, years (SD)	72.34 (16.73)	44.88 (20.46)	< 0.001
Gender (% male)	829 (42.0)	1112 (68.2)	< 0.001
ISS, mean (SD)	9.96 (4.21)	14.01 (9.29)	< 0.001
Type of fracture			
Femoral neck fracture	931 (47.1)	NA	
Trochanteric fracture	1044 (52.9)	NA	

*SD* standard deviation, *ISS* injury severity score

**Fig. 1:**
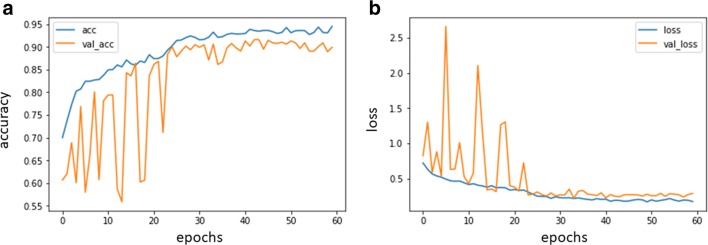
Performance in the training and validation datasets using TensorBoard. a. Accuracy change during training process. acc, accuracy of the training set; val_acc, accuracy of the validation set;b. Change of loss during training process. loss, loss of the training set; val_loss, loss of the validation set

The model was trained with a limb radiograph dataset and finally showed an accuracy of 99.5% on the validation dataset for identifying specific body parts. The weights in the limb model were preserved and named the “limb pretrained model.” The model was retrained again using the weights from the limb pretrained model, and the task was changed to detect hip fractures. The final training accuracy values for the training set was 0.94 and for the validation set was 0.90, respectively. The change of accuracy and loss during the training process are shown in Fig. 1.

**Fig. 2:**
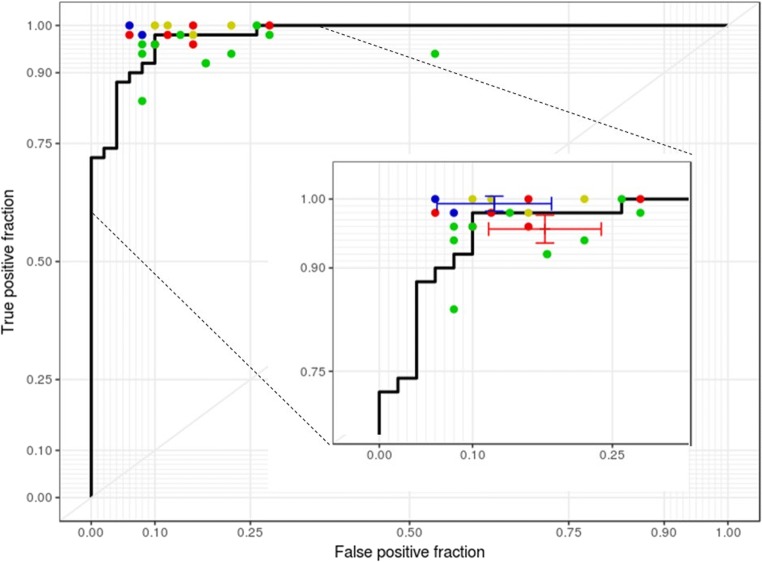
Performances of the hip model and physicians. The blue, green, yellow, and red spots indicate the performance of radiologists, surgeons, orthopedic doctors, and emergency physicians, respectively

After applying the hip model to the 2017 PXR dataset, the accuracy, sensitivity, specificity, false-negative rate, and F1 score of the model were 91% (*n* = 100; 95% CI, 84–96%), 98% (95% CI, 89–100%), 84% (95% CI, 71–93%), 2% (95% CI, 0.3–17%), and 0.916 (95% CI, 0.845–0.956), respectively. A total of 21 experts completed the questionnaire. The range of sensitivity of primary physicians (except radiologists and orthopedic surgeons) was 84% to 100% (mean, 95.6%; 95% CI, 93.6–97.6%), and the specificity ranged from 46 to 94% (mean, 82.2%; 95% CI, 76.2–88.3%). The experts, including two radiologists and four orthopedic surgeons, completed the questionnaire and achieved a mean sensitivity of 99.3% (95% CI, 98.2–100%) and a specificity of 87.7% (95% CI, 81.5–93.8%). The ROC curve of prediction probability compared with individual specialist performance is shown in Fig. 2. The model achieved an AUC of 0.98 (95% CI, 0.96–1.00). The 95% CI of both mean sensitivity and mean specificity of the primary physicians were below the ROC curve of the model, and the mean performance of radiologists and orthopedic surgeons was still slightly better than that of the model.

**Fig. 3:**
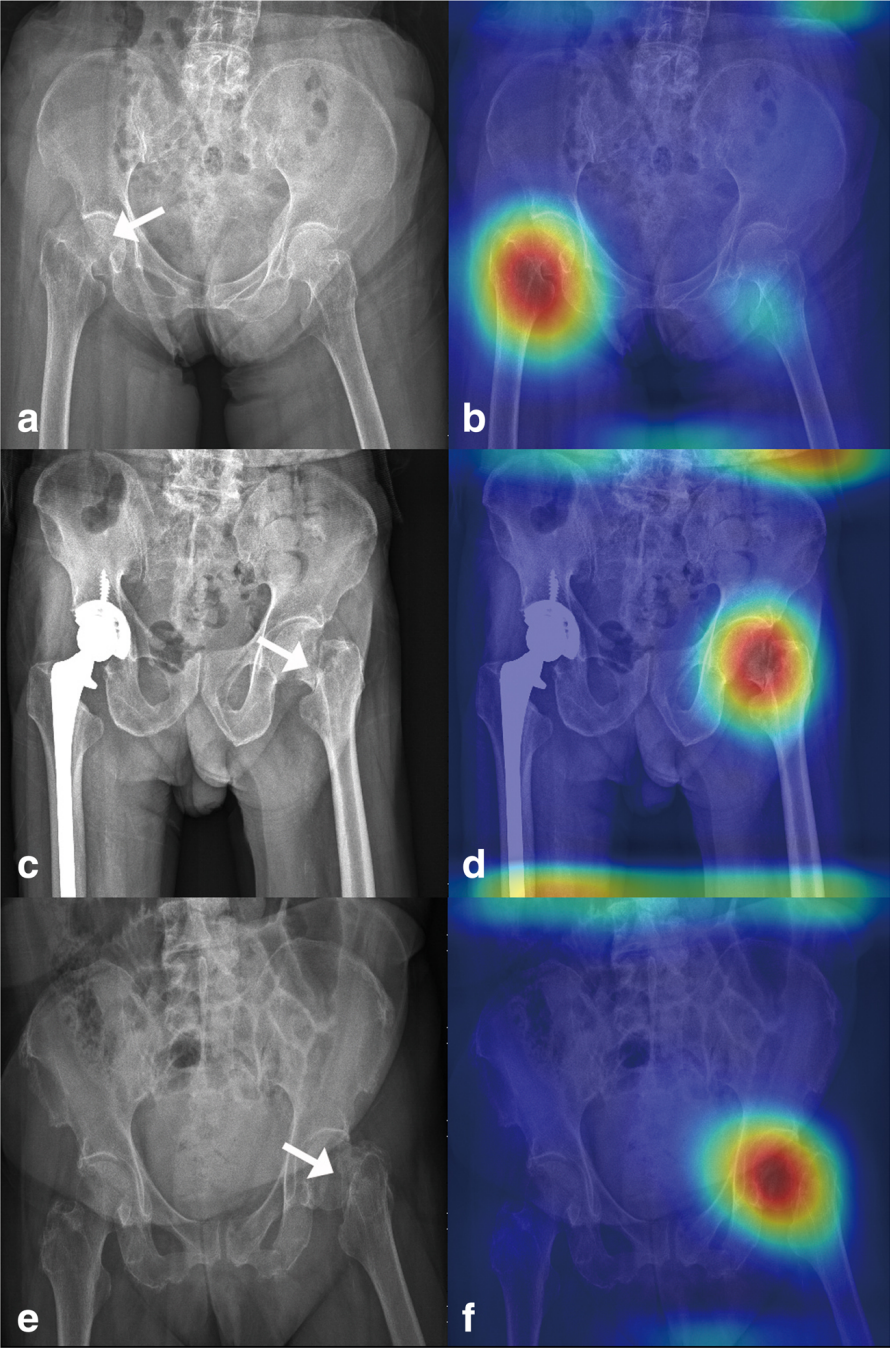
Grad-CAM-assisted image identification of hip fractures. **a** The original pelvic radiograph with a mildly displaced right femoral neck fracture (arrow) and **b** the image generated after applying the model with Grad-CAM, which visualizes the class-discriminative regions, as the fracture site. **c** PXR presenting a right total hip replacement with a left femoral neck fracture (arrow) and **d** a Grad-CAM-assisted image. **e** PXR with a mildly displaced left femoral neck fracture and **f** a Grad-CAM-assisted image

After analyzing 49 heatmap images, the model predicted positive hip fracture. Only two images identified the wrong activation site, and 95.9% of the activation area was located at the hip fracture site, as shown in Fig. 3.

## Discussion

These results indicate that a DCNN can be trained to identify hip fractures within image datasets with high sensitivity (98%) and accuracy (91%) (F1 score, 0.91). Currently, hip fractures occur daily, and uneven or nondisplaced hip fractures are challenging to rapidly identify because of the limitations of the human eye and PXRs [14, 15]. With the assistance of the DCNN, we can detect hip fractures immediately with a low false-negative rate (0.02), which is noninferior to the performance of the experts. This DCNN will be useful for primary physicians to lessen the misdiagnosis rate and to prevent subsequent misdiagnosis events. Hip fractures are a promising target for deep learning approaches because of the availability of near-perfect ground truth labels. Because of the weight-bearing nature of the region, patients who have clinically “silent” fractures rapidly develop severe pain and immobility. Early detection and surgery are critical for patient survival and the preservation of hip function. Postponed management of hip fractures results in a poor prognosis and even an increased risk of death years later [35–38]. Therefore, detecting hip fractures as soon as possible is critical for remote mortality and medical outcomes.

DCNNs allow computers to learn from iterations with automatic feature extraction under limited programming, and the prediction rate is highly accurate. Artificial intelligence and the automation of bony fracture detection have been discussed [25, 26, 28]; Gale et al also described a DCNN method that was used for predicting hip fractures [29]. However, the model developed previously for hip fractures is a region base model and requires a localization network to identify the femoral neck first. Our study shows that the detection and diagnosis of hip fractures on PXRs could be performed by the input of a whole-scale radiograph to a DCNN without identifying the specific region first. The deep learning algorithm also achieved an accuracy level that is compatible with the accuracy of radiologists and orthopedic surgeons. Moreover, this study also substantially added to other current studies.

First, we applied a transferring learning method to develop our algorithm. We set the pretrained model using 25,505 unlabeled limb radiographs instead of the Image-Net images because we believe that a pretrained model using similar images reduces the required image sample size and training time. This study does not compare performance between the Image-Net pretrained model and the limb pretrained model. We entered 2804 frontal PXRs as training material. After evaluation, our accuracy increased from 79% (scratch pretrained) to 91% (limb dataset pretrained), as we expected, and the pretraining material also impacted the final accuracy.

Unlike in previous works, the process did not require much extensive processing, lesion segmentation, or extraction of domain-specific visual features. In contrast, our system needs no handcrafted features, and it is trained end to end directly from image labels and raw pixels. Our fully automated system takes PXRs and automatically detects the presence of hip fractures. These results demonstrate that deep neural networks can be trained using sizable non-pixel labeled datasets without having to specify lesion-based features. Our research shows that despite the challenges specific to radiographic data, the development of large, clean datasets is sufficient to achieve high-level automated performance with deep learning systems. In this way, we can save the time associated with segmentation and labeling.

**Fig. 4:**
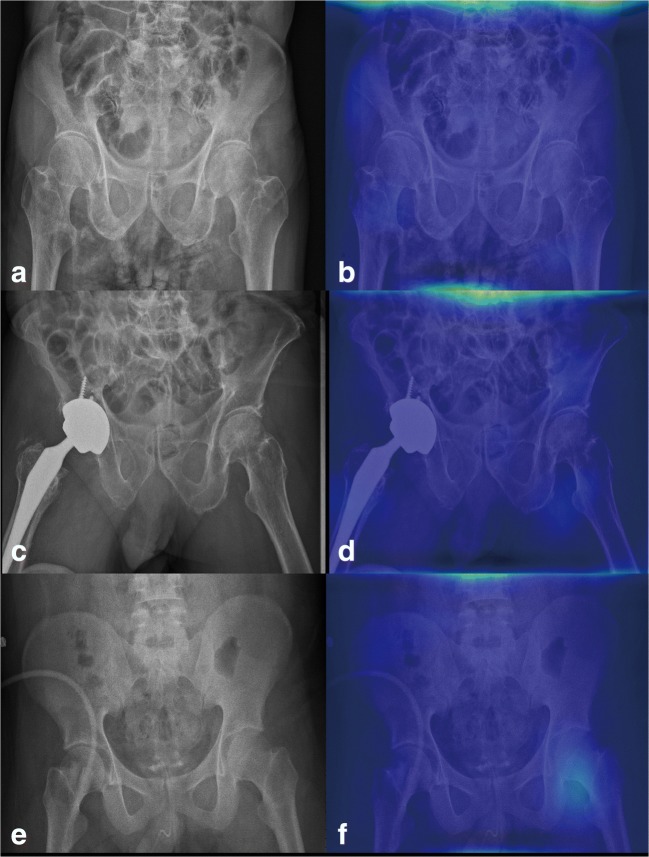
Grad-CAM-assisted image of a normal PXR. **a** Frontal PXR without a hip fracture and **b** a Grad-CAM image with no heatmap. **c** Frontal PXR with a right hip replacement without a hip fracture and **d** a Grad-CAM image showing no identification of a fracture site. **e** Frontal PXR without a hip fracture and **f** Grad-CAM visualized no class-discriminative regions

One paradox in DCNNs for analyzing medical images is the “black box” mechanism. The model may use another part of the image rather than the true lesion site to produce the answer. Therefore, visualization of the features became a solution to realizing the underlying mechanism of DCNNs [39]. In this study, we used whole PXRs for training and testing. Then, we performed Grad-CAM to visualize the class-discriminative regions as the fracture sites that DCNN recognizes in the PXRs (Fig. 3). On the other hand, in normal PXRs, the Grad-CAM films lacked a heatmap (Fig. 4). After visualization by the Grad-CAM method, 95.9% of the class-discriminative regions contained the fracture site, which provided evidence that the model indeed recognized the hip fracture. To understand what a DCNN uses to make predictions is a very active research topic in medical aspects, and it may convince doctors to accept the results because the DCNN can explain what they find.

Most deep learning works evaluating medical images use cropped images to avoid “black box” mechanisms and enhance the accuracy of final validation [29, 32]. Once the target is cropped to include the necessary features for recognition, the DCNN will detect the lesion more easily and quickly. In this study, instead of cropping images, we reduced the image matrix size to 512 × 512 pixels. We prefer to input whole PXR images because this method might be more instinctual and physicians are more familiar with it. Because we integrated the DCNN into the clinical pathway, doctors will prefer to use whole images rather than cropped images. The dimensionality reduction also decreases the computational requirement and shortens the training time with an acceptable result. Furthermore, based on our model, we also applied this algorithm to other types of fracture in PXRs. In the future, based on the observations from this study, the development of similar high-performance algorithms for medical imaging might shorten the training process time and resources required.

This study has several limitations. One fundamental limitation arises from the nature of DCNNs because the neural network was provided with only an image and associated diagnosis, without explicit definitions of features. Because the DCNN “learned” the features that were most predictive, the algorithm might use features previously unknown to or ignored by humans. Although this study showed good visualization for identifying fractures, the exact features being used are still unknown. It is possible that the heatmaps show differences in femoral alignment or soft tissue contrast due to edema or hemorrhage between the fractured and nonfractured sites. In this study, two of the images did not demonstrate the right activation location of the fracture. One of the wrong activation sites is located on the opposite hip, and another had a stronger signal over the iliac bone region. This error is indeed a limitation of DCNN because it is difficult to explain why it activates at the wrong site. Inputting the whole image to the model is also the most challenging part, and we will exert further effort to solve this issue using a greater number of input samples. In addition, the algorithm was specifically trained to discriminate between healthy bones and fractures in the background of the bony architecture on radiographs, but the algorithm might be unable to identify other pathological presentations. The detection of other lesions on PXRs, which is relevant for routine diagnoses, was not included in this study. In the current study, although we used only PXR as the input material, we still observed differences in age, gender, and ISS between both fracture and nonfracture patients, which might create some selective bias. Furthermore, we extracted the hip fracture images by final diagnosis from the registry and identified the fracture site on the images when we validated the performance of the DCNN. Therefore, it is difficult to clarify the exact occult fracture rate. In this study, we still manually excluded a certain proportion of images, which will be problematic when trying to incorporate this process into the clinical workflow. Finally, although the results of this study are promising, integrating this automatic detection algorithm into clinical work to increase the detection rate of hip fractures presents another challenge. Therefore, we utilized a web-based system that can input the PXR from PACS in the hospital, and the DCNN will detect the presence of a fracture or not and localize the fracture region by Grad-CAM. A randomized, prospective study should be conducted to evaluate the clinical impact on the diagnostic accuracy and economic value of DCNN for identifying hip fractures.

In conclusion, to identify hip fractures on PXRs, the algorithm trained by a DCNN achieved excellent performance with a high accuracy and low false-negative rate and is useful as a screening tool. Furthermore, our algorithm can localize the fracture site with high accuracy and thus assist clinical physicians in identifying more occult hip fractures and managing patients with these fractures early to prevent further medical costs and decreased quality of life.

## Abbreviations

AUC: Area under the curve
CAM: Class activation mapping
CGMH: Chang Gung Memorial Hospital
CGTRP: Chang Gung Trauma Registry Program
CT: Computed tomography
DCNN: Deep convolutional neural network
Grad-CAM: Gradient-weighted class activation mapping
MRI: Magnetic resonance imaging
PXR: Frontal pelvic radiograph
ROC: Receiver operating characteristic
